# Essential interventions for child health

**DOI:** 10.1186/1742-4755-11-S1-S4

**Published:** 2014-08-21

**Authors:** Zohra S Lassi, Dania Mallick, Jai K Das, Lekho Mal, Rehana A Salam, Zulfiqar A Bhutta

**Affiliations:** 1 Division of Women and Child Health, Aga Khan University, Karachi, Pakistan

**Keywords:** essential interventions, child health, children

## Abstract

Child health is a growing concern at the global level, as infectious diseases and preventable conditions claim hundreds of lives of children under the age of five in low-income countries. Approximately 7.6 million children under five years of age died in 2011, calculating to about 19 000 children each day and almost 800 every hour. About 80 percent of the world’s under-five deaths in 2011 occurred in only 25 countries, and about half in only five countries: India, Nigeria, Democratic Republic of the Congo, Pakistan and China. The implications and burden of such statistics are huge and will have dire consequences if they are not corrected promptly. This paper reviews essential interventions for improving child health, which if implemented properly and according to guidelines have been found to improve child health outcomes, as well as reduce morbidity and mortality rates. It also includes caregivers and delivery strategies for each intervention. Interventions that have been associated with a decrease in mortality and disease rates include exclusive breastfeeding, complementary feeding strategies, routine immunizations and vaccinations for children, preventative zinc supplementation in children, and vitamin A supplementation in vitamin A deficient populations.

## Introduction

In the past few years, an inordinate amount of effort has been put in towards the achievement of the millennium development goal (MDG) for child survival- MDG 4. Though some countries are on MDG target to reduce under-five mortality by two-thirds, the total number of child deaths has actually increased in 13 countries [1]. On a more positive note, under-five mortality worldwide has decreased from approximately 9.6 million in 2000 to 7.6 million in 2012 [2]. The death of 19,000 children under age five every day in 2011 [2] is still a major concern, which dictates that evaluations are necessary for further improvement.

Around half of under-five deaths occur in only five countries: India, Nigeria, Democratic Republic of Congo, Pakistan and China [2]. The leading causes of death among children under age five are pneumonia (18% of all under-five deaths); preterm birth complications (14%); diarrhea (11%); intrapartum related complications (complications during birth; 9%); and malaria (7%) [2]. Most of these complications are preventable with the correct interventions being implemented. Another major factor leading to the inability to meet proposed reduction in figures of child mortality is the fact that most maternal and child health programs do not reach the world’s poorest families [3]. For children who need them the most, survival interventions such as vaccination programs have showed incomplete coverage. Some programs have shown tremendous success, such as the increased coverage of measles vaccination, which have resulted in a 92% reduction in measles mortality between 2000 and 2008 in sub-Saharan Africa [2]. Problems associated with lack of coverage include poorly designed health care systems in the developing world where a lack of resources including skilled workers are a major concern [3]. In some low and middle income countries (LMICs), such as Pakistan or India, resistance or refusals against vaccines that are deep rooted in social-cultural, religious and political contexts as well as gender-based decision-making in households are direct causes of the high rates of disease burdens such as polio [4].

The aim of this review is to underscore the effectiveness of essential child health interventions that have an alleged impact on reducing child illnesses and improving child survival and can be suitably delivered to resource limited countries.

## Methodology

The methodology has been described in detail elsewhere [4]. In short, the review included all child health interventions based on current World Health Organization (WHO) guidelines and recent Lancet series which have an alleged impact on reducing child morbidity and mortality; suitable for delivery in LMICs; and those that can be delivered through the health sector (community level up to the referral level of health care) (Table 1). All relevant childbirth and postnatal intervention reviews were identified from the electronic databases such as the Cochrane database of systematic reviews, the Cochrane database of abstract reviews of effectiveness (DARE), the Cochrane database of systematic reviews of randomized control trials (RCT’s), and PubMed. The reference lists of the reviews and recommendations from experts in the field were also used as sources to obtain additional publications. The principal focus was on the existing systematic reviews and meta-analysis. Based on the efficiency of the interventions, these were then classified in categories from A to E (where A signified strongly beneficial effect while E indicated harmful effect) on different levels of health sector (community/outreach/referral).

**Table 1:** Characteristics of the included reviews on child health interventions

Reviews	Objective	Type of Studies included (number)	Cochrane/non-Cochrane	Pooled Data (Y/N)	Outcomes reported
**Imdad 2011 **[8]	To assess the effectiveness of breastfeeding promotion interventions on breastfeeding rates in early infancy.	RCTs and qRCTs: 53	Non-Cochrane	Yes	EBF at 4-6 weeks postpartum

**Dyson 2005 **[9]	To evaluate the effectiveness of interventions which aim to encourage women to breastfeed in terms of changes in the number of women who start to breastfeed.	RCTs: 7	Cochrane	Yes	Increasing breastfeeding initiation rates

**Haroon 2013 **[10]	A systematic literature search was conducted for RCTs and quasi-experimental studies comparing breastfeeding education or support to routine care. The	RCTs: 21	Non-Cochrane	Yes	Exclusive breastfeeding rates

**Kramer 2012 **[16]	To assess the effects on child health, growth, and development, and on maternal health, of exclusive breastfeeding for six months versus exclusive breastfeeding for three to four months with mixed breastfeeding (introduction of complementary liquid or solid foods with continued breastfeeding) thereafter through six months.	RCTs: 2 Observational studies: 18	Cochrane	Yes	Exclusive breastfeeding rates

**Lassi 2013 **[20]	The effect of complementary feeding (CF) (fortified or unfortified, but not micronutrients alone) and education on CF on children less than 2 years of age in low and middle income countries (LMIC).	RCTs and qRCTs: 16	Non-Cochrane	Yes	HAZ, WAZ, stunting

**Bhutta 2008 **[21]	The effect of complementary feeding (CF) in food secure and food insecure population	RCTs: 7	Cochrane	Yes	HAZ, WAZ, stunting

**Lengler 2004 **[22]	To assess the impact of insecticide-treated bed nets or curtains on mortality, malarial illness (life-threatening and mild), malaria parasitaemia, anaemia, and spleen rates.	RCTs: 14	Cochrane	Yes	Mortality, malarial illness (life-threatening and mild), malaria parasitaemia, anaemia, and spleen rates.

**Meremikwu 2012 **[24]	To evaluate the effects of IPTc to prevent malaria in preschool children living in endemic areas with seasonal malaria transmission.	RCTs: 7	Cochrane	Yes	Clinical malaria episode, all-cause mortality

**Thwing 2011 **[25]	We performed systematic literature reviews of published studies in P. falciparum endemic settings to determine the protective efficacy (PE) of ACT treatment against malaria deaths among children with uncomplicated malaria, as well as the PE of effective case management including parenteral quinine against malaria deaths among all hospitalized children.	Ata sources	Non-Cochrane	Yes	Malaria mortality

**Eisele 2010 **[26]	To estimate the effect of ITNs and IRS on preventing malaria-attributable mortality in children 1–59 months, and to estimate the effect of ITNs and IPTp on preventing neonatal and child mortality through improvements in birth outcomes.	RCTs: 14	Non-Cochrane	Yes	Protective efficacy, malaria-attributable mortality 1–59 months, prevention interventions in pregnancy

**Mathanga 2011 **[27]	To compare intermittent preventive treatment regimens for malaria in HIV-positive pregnant women living in malaria-endemic areas.	RCTs: 2	Cochrane	Yes	Maternal anaemia, low birth weight, and neonatal mortality

**Grimwade 2006 **[28]	To assess the effects of routinely administered cotrimoxazole on death and illness episodes in children with HIV infection, and in infants of HIVinfected mothers.	RCTs: -	Cochrane	No	-

**Siefried 2011 **[30]	To determine whether, and to what extent, antiretroviral regimens aimed at decreasing the risk of mother-to-child transmission of HIV infection achieve a clinically useful decrease in transmission risk, and what effect these interventions have on maternal and infant mortality and morbidity.	RCTs: 25	Cochrane	Yes	Reduction in the proportion infected:

**Adeitfa 2009 **[31]	To assess the effects of routinely administered cotrimoxazole on death and illness episodes in children with HIV infection, and in infants of HIV infected mothers.	RCTs and qRCT: 0	Cochrane	No	-

**Chetty 2010 **[84]	The objective of the systematic review was to pool and evaluate the data on the effectiveness of different infant feeding practices from birth to 18months in achieving HIV-free survival of HIV-exposed infants.	RCTS:17 Observational studies: 17 Secondary articles: 18	Non-Cochrane	Yes	Mixed breastfeeding/replacement feeding up to 6 months of life

**Soares-Weiser 2012 **[34]	To evaluate rotavirus vaccines approved for use (Rotarix, RotaTeq, and Lanzhou Lamb Rotavirus (LLR)) for preventing rotavirus diarrhoea.	RCTs: 34	Cochrane	Yes	Rotavirus diarrhoea, all-cause diarrhoea (severe cases), and hospitalizations and need for medical attention

**Soares-Weiser 2004 **[35]	To assess rotavirus vaccines in relation to preventing rotavirus diarrhoea, death, and adverse events.	RCTs: 64	Cochrane	Yes	Rotavirus diarrhoea, all-cause diarrhoea (severe cases), and hospitalizations and need for medical attention

**Munos 2010 **[36]	To assess efficacy and effectiveness trials of rotavirus vaccines	RCTs: 25	Non-Cochrane	Yes	Hospitalizations

**Das 2013 **[37]	To assess efficacy and effectiveness trials of vaccines	RCTs and qRCTs: 24	Non-Cochrane	Yes	Mortality, rota-virus specific moetality

**Imdad 2011 **[42]	The purpose of this paper was to get a point estimate of efficacy of vitamin A supplementation in reducing cause specific mortality by using Child Health Epidemiology Reference Group (CHERG) guidelines.	RCTs: 21	Non-Cochrane	Yes	All-cause mortality, diarrhea specific mortality, meningitis, and pneumonia specific mortality

**Imdad 2010 **[43]	To evaluate the effect of vitamin A supplementation (VAS) for preventing morbidity and mortality in children aged 6 months to 5 years.	RCTs: 43	Cochrane	Yes	All-cause mortality, diarrhea specific mortality, meningitis, and pneumonia specific mortality

**Mayo-Wilson 2011 **[44]	To determine if vitamin A supplementation is associated with reductions in mortality and morbidity in children aged 6 months to 5 years.	RCTs: 43	Cochrane	Yes	All-cause mortality, diarrhea specific mortality, meningitis, and pneumonia specific mortality

**Ahmed 2010 **[46]	To evaluate the management of severe acute malnutrition according to WHO guidelines	RCTs: 25	Non-Cochrane	Yes	Mortality, weight gain

**Lenters 2013 **[47]	To evaluate the effectiveness of interventions for SAM including the World Health Organization (WHO) protocol for inpatient management and community-based management with ready-to-use-therapeutic food (RUTF), as well as interventions for MAM in children under five years in low- and middle-income countries.	RCTs: 14	Non-Cochrane	Yes	Case fatality

**Theodaratou 2010 **[49]	To assess the effect of pneumonia case management on mortality from childhood pneumonia.	RCTs: 25	Non-Cochrane	Yes	All-cause mortality, pneumonia specific mortality

**Sazawal 2003 **[50]	This meta-analysis provides estimates of mortality impact of the case-management approach proposed by WHO.	RCTs: 7	Non-Cochrane	Yes	All-cause mortality, pneumonia specific mortality

**Das 2013 **[51]	To estimate the effect of community based interventions including community case management on the coverage of various commodities and on mortality due to diarrhea and pneumonia.	RCTs and qRCTs: 24	Non-Cochrane	Yes	Care seeking for pneumonia and diarrhea, treatment failure, case management

**Fawzi 1993 **[52]	A two-part meta-analysis of studies examining the relationship of vitamin A supplementation and child mortality.	RCTs: 12	Non-Cochrane	Yes	All-cause mortality,

**Sudfeld 2010 **[53]	To determine effect estimates of measles vaccine and vitamin A treatment for the Lives Saved Tool (LiST).	RCTs and qRCT: 525	Non-Cochrane	Yes	Preventing measles disease

**Brown 2004 **[54]	To determine the efficacy of intervention with high-dose vitamin A as an adjunct to standard treatment on outcome in acute lower respiratory tract infection in children in developing countries.	RCTs: 5	Non-Cochrane	Yes	Faster recovery; oxygen requirement; raised respiratory rate; hospital stay, mortality

**Wu 2005 **[55]	To determine whether adjunctive vitamin A is effective in children diagnosed with non-measles pneumonia.	RCTs: 6	Cochrane	Yes	Mortality, hospital stay

**Grotto 2003 **[56]	To perform an updated meta-analysis of the effect of vitamin A supplementation on childhood morbidity from respiratory tract infections and diarrhea.	RCTs: 9	Non-Cochrane	Yes	Incidence of diarrhea, incidence of respiratory tract infections

**Chen 2008 **[57]	To assess the effectiveness and safety of vitamin A for preventing acute LRTIs in children up to seven years of age.	RCTs: 10	Cochrane	Yes	Incidence of acute LRTI in one study; an increase in cough and fever; and increased symptoms of cough and rapid breathing

**Lamberti 2013 **[58]	To quantify the protective effects of breastfeeding exposure against pneumonia incidence, prevalence,hospitalizations and mortality	Prospective cohort= 7 Case-control = 3	Non-Cochrane	Yes	Pneumonia mortality

**Yakoob 2011 **[62]	To determine all-cause mortality and cause-specific mortality and morbidity in children under five in developing countries for preventive zinc supplementation.	RCTs: 8	Cochrane	Yes	Diarrhea-specific mortality and pneumonia-specific mortality

**Lazzirini 2013 **[66]	To evaluate oral zinc supplementation for treating children with acute or persistent diarrhoea	RCTs: 18	Cochrane	Yes	Diarrhea duration

**Gregorio 2009 **[67]	To compare polymer-based ORS with glucose-based ORS for treating acute watery diarrhoea.	RCTs: 34	Cochrane	Yes	Unscheduled intravenous infusions, duration of diarrhea

**Hartling 2006 **[68]	To compare oral with intravenous therapy for treating dehydration due to acute gastroenteritis in children.	RCTs: 17	Cochrane	Yes	Intravenous infusions, duration of diarrhea, hospital stay, oral intake

**Hahn 2002 **[69]	To compare reduced osmolarity oral rehydration solution with the World Health Organization recommended strength for treating diarrhoea in children.	RCTs: 41	Cochrane	Yes	Unscheduled intravenous infusions

**Lenters 2013 **[70]	To understand which interventions are effective in promoting the use of ORS, and where there are gaps in the literature	RCTs: 19	Non-Cochrane	Yes	Diarrhea episodes

**Das 2013 **[71]	To estimate the effect of antiemetics in gastroenteritis in children	RCTs: 7	Non-Cochrane	Yes	Incidence of vomiting and hospitalization

**Christopher 2010 **[72]	To evaluate the efficacy and safety of antibiotics for treating Shigella dysentery.	RCTs: 16	Cochrane	Yes	Incidence of diarrhea

**Traa 2010 **[73]	To review the effect of ciprofloxacin, ceftriaxone and pivmecillinam for the treatment of dysentery in children in the developing countries.	RCTs: 19	Non-Cochrane	Yes	Rates of treatment failure, bacteriological failure and bacteriological relapse

**Das 2013 **[74]	To review the literature reporting the effect of antibiotics for the treatment of diarrhea due to cholera, Shigella and Cryptosporidium in children under five years	RCTs: 6	Non-Cochrane	Yes	Mortality and cause specific mortality

**Musekiwa 2011 **[77]	To compare the safety and efficacy of ORS ≤270 with ORS ≥ 310 for treating dehydration due to cholera.	RCTs: 7	Cochrane	Yes	Biochemical hyponatraemia

## Results

### Promotion of breastfeeding

Evidence suggests that the safest and most successful way to assure proper growth and development in child is by providing the infant with breast milk from the first hour of birth [5-7]. Exclusive breastfeeding also protects the newborn from various diseases such as respiratory infections and diarrhea, which can lead to insidious outcomes for the newborn. Even though these protective effects of breastfeeding have been stated for years, the duration of exclusive breastfeeding is still far below its optimal level it should be in most countries, both high income countries (HICs) and LMICs. This review assessed the interventions that provided counseling on the benefits of exclusive breastfeeding in the neonatal period and at six months to all pregnant mothers, either individually or in groups.

A review found increased rates of exclusive breastfeeding in neonatal period (risk ratio (RR) 3.06; 95% confidence interval (CI): 2.10, 4.45) as well as at 6 months (RR 1.86; 95%CI: 1.12, 3.09) on comparing individual counseling with routine care [8]. Group counseling, on the other hand, resulted in higher exclusive breastfeeding rates both during neonatal period (RR 3.95; 95%CI: 2.09, 7.44) and at 6 months (RR 4.49; 95%CI: 1.90, 10.63) [8]. Other interventions for improving exclusive breastfeeding included providing health education for women of all feeding intentions vs. standard care (RR 1.57; 95% CI: 1.15, 2.15), peer support for women considering breastfeeding (RR 4.02; 95% CI: 2.63, 6.14) and breastfeeding promotion packs for women of all feeding intentions (RR 0.93; 95% CI: 0.80, 1.08) [9].

Similar findings have been reported in a recent review which indicated that educational interventions significantly increased exclusive breastfeeding rates (EBF) rates at day 1 by 43% (RR 1.43; 95% CI: 1.09, 1.87), at <1 month by 30% (RR 1.30; 95% CI: 1.19, 1.42) and at 1-5 months by 90% (RR 1.90; 95% CI: 1.54, 2.34) [10]. Subgroup analyses showed that individual counseling alone led to a 60% increase in exclusive breastfeeding rates (RR 1.60; 95% CI: 1.04, 2.48) [10].

### Promotion and support of continued breastfeeding and complementary feeding

a) Continued breastfeeding

Growth faltering is a commonly observed phenomenon in LMICs after about three months of age [11-13]. This growth faltering has traditionally been attributed to three factors: (1) the suggested inadequacy of energy intake from breast milk alone after three or four months; (2) the poor nutritional quality (i.e., low energy and micronutrient content) of the weaning foods commonly introduced in many developing countries; and (3) the adverse effects of infection on energy intake and expenditure. The belief that breast milk alone is nutritionally insufficient after three or four months, combined with the fact that weaning foods given in many LMICS are both nutritionally inadequate and contaminated, led to concern about the so-called ’weanling’s dilemma’ [14,15]. Because breastfeeding can be lifesaving in LMICs and the health benefits of breastfeeding are widely acknowledged, opinions and recommendations are strongly divided on the optimal duration of exclusive breastfeeding.

A recent Cochrane review [16] found that infants who are exclusively breastfed for six months experience less morbidity from gastrointestinal infection than those who are partially breastfed as of three or four months (RR 0.41; 95% CI: 0.21, 0.78), and no deficits have been demonstrated in growth among infants from either developing or developed countries who are exclusively breastfed for six months or longer. Moreover the results from controlled trials showed that exclusive versus mixed breast-feeding in developing countries resulted in a monthly weight gain from 4-6 months (MD 20.78g/month; 95% CI: -21.99, 63.54); and from 6-12 months (MD -2.62g/month; 95% CI: -25.85, 20.62) and a hemoglobin concentration of <110 g/dl (RR 1.20; 95% CI: 0.90, 1.58). The same review using observational studies showed a monthly weight gain from 4-6 months (MD -10.10; 95% CI: -27.68, 7.48) and from 6-9 months (MD -6.0; 95% CI: -54.15, 42.15).

b) Education strategies on complementary feeding support

Period of complementary feeding (CF) (from 6 to 24 months of age) is one of the most critical times for preventing malnutrition [17]. Effect on growth is most evident during this time [11] particularly during the first phase of complementary feeding when foods of low nutrient density begin to replace breast milk and rates of diarrheal illness caused by food contamination are at their highest. This is consistent with results of intervention trials showing that the greatest impact of food supplementation is seen among children less than 2 years of age [18,19]. CF is a food-based intervention, given at the age range of 6-24 months, along with breastfeeding to fulfill the extra needs of the growing infant. Several strategies can be used for promotion of this intervention, such as education about CF as the main treatment, and provision of complementary foods.

A recent review has reported that education on CF alone significantly improved height-for-age Z score (HAZ) (standard mean difference (SMD): 0.23; 95% CI: 0.09, 0.36), weight-for-age Z score (WAZ) (SMD 0.16, 95% CI: 0.05, 0.27), and significantly reduced the rates of stunting (RR 0.71; 95% CI: 0.56, 0.91, 5 studies); no significant impact were observed for height and weight gain [20]. However, based on the subgroup analysis; studies from food secure populations indicated education on CF had a significant impact on height gain (SMD 0.35; 95% CI: 0.08, 0.62), HAZ scores (SMD 0.22; 95% CI: 0.01, 0.43), and weight gain (SMD 0.40; 95% CI: 0.02, 0.78), however, stunting reduced non-significantly (RR 0.70; 95% CI: 0.49 1.01) [20]. On the other hand, in food insecure population, CF education alone significantly improved HAZ scores (SMD 0.25; 95% CI: 0.09, 0.42), WAZ scores (SMD 0.26; 95% CI: 0.12, 0.41) and significantly reduced the rates of stunting (RR 0.68; 95% CI: 0.60, 0.76), while CF provision with or without education improved HAZ (SMD 0.39; 95% CI: 0.05, 0.73) and WAZ scores (SMD 0.26; 95% CI: 0.04, 0.48) significantly. Similar results were also reported in an earlier Lancet review [21].

### Prevention and management of childhood malaria

a) Provision and promotion of use of insecticide treated nets (ITNs) for children

ITNs not only provide a physical barrier to bites from mosquitoes, but their insecticide component like pyrethroids also repel and kill them.

A Cochrane review reported 17% protective efficacy (PE) (RR 0.83, 95% CI 0.76, 0.90) with ITNs when compared with no nets and 23% PE compared to untreated nets (RR 0.77, 95% CI 0.63, 0.95) and about 5.5 lives (95% CI 3.39, 7.67) can be saved each year for every 1000 children protected with ITNs [22]. It further reported 18% reduction in all-cause child mortality (RR 0.82; 95% CI: 0.76, 0.89) [22].

b) Malarial prophylaxis in children:

Intermittent preventive treatment or intermittent presumptive treatment (IPT) involves giving the drug e.g. sulfadoxine-pyrimethamine, at full therapeutic dose to the whole population at risk at specific times to prevent mortality or morbidity [23].

IPT for malaria in children living in areas with seasonal transmission showed lower rates of clinical malaria during intervention (RR 0.26; 95% CI: 0.17, 0.38), and during post-intervention (RR 0.98; 95% CI: 0.82, 1.17) [24]. The same review found lower cases of severe malaria during intervention (RR 0.27; 95% CI:0.10, 0.76), and lower rates of parasitaemia malaria during intervention (RR 0.35; 95% CI: 0.25, 0.50) as well as during post-intervention (RR 1.01; 95% CI: 0.89, 1.16) [24]. Artemisinin-based combination therapies also showed significant results on reducing malaria in children 1-23 months of age (RR 0.01; 95% CI: 0.00, 0.06); and children 24-59 months (RR 0.03; 95% CI: 0.01, 0.14) [25]. The review also estimated the PE of treatment of severe *P. falciparum* malaria with effective case management including intravenous quinine on reducing malaria mortality in children 1-59 months to be 82% (range: 63-94%) compared to no treatment [25]. Another review estimated 55%PE with ITNs and indoor-residual spray on reducing malaria-attributable mortality in children 1-59 months (RR 0.45; 95% CI: 0.39, 0.51) [26].

The use of monthly sulfadoxine-pyrimethamine (SP) compared to a standard 2-dose SP in HIV-positive pregnant women, resulted in non-significant reduction in maternal anemia (Hb<11g/dl) (RR 0.97, 95% CI0.84, 1.12), however, presence of malaria in the placental blood (placental parasitaemia) decreased (RR 0.42; 95% CI: 0.23, 0.76) [27]. No improvements were observed in in neonatal mortality (RR 0.29; 95% CI: 0.08, 1.05) [27].

### Comprehensive care of children infected or exposed to HIV infection

HIV infection in children (not on antiretroviral therapy (ART)) follows a more destructive course, with death occurring in about 30% by the 1^st^ year and 50% by the age of 2 years [28,29]. Prophylaxis with co-trimoxazole is a drug of choice for infants who are born to HIV infected mothers, since the detection of the HIV from birth till 18 months is very difficult due to the presence of maternal HIV antibodies.

The results of the Cochrane review on administration of cotrimoxazole prophylaxis vs. placebo found a single trial from Zambia and the study reported reduction in child mortality (RR 0.67; 95% CI: 0.53, 0.85), and hospital admission (RR 0.77; 95%CI: 0.62, 0.96) [28]. Zidovudine (ZDV) plus lamivudine (3TC) given to mothers from 36 weeks gestation, during labor and for 7 days after delivery and to babies for the first 7 days after birth (PETRA 'regimen A') significantly reduced HIV infection (Efficacy 62.75%; 95% CI: 40.76, 84.74) [30].

Iron supplementation may have a role in reducing mortality and morbidity in HIV infected children, but no randomized controlled trials on the subject were found [31].

### Promote and provide routine immunization and vaccinations in infants/children

Expanded Program on Immunization (EPI) schedule for poliomyelitis, diphtheria, pertussis, tetanus, measles and tuberculosis is one of the most cost-effective ways to improve child health [32]. It has been estimated that over 3 million deaths are averted each year from measles, pertussis and neonatal tetanus by vaccination [33]. Rotaviruses cause viral gastroenteritis and result in more deaths from diarrhea in children under 5 years of age than any other single agent, particularly in LMICs [34]. The WHO recommends that all children should be vaccinated with a monovalent rotavirus vaccine or a pentavalent rotavirus vaccine, with a stronger recommendation for countries where deaths due to diarrhea comprise more than 10% of all deaths [34]. Immunization against Haemophilus influenzae type b (Hib) is recommended where resources permit its use and the burden of disease is established. WHO considers that pneumococcal conjugate vaccine should be a priority for inclusion in national childhood immunization programs. Countries with mortality among children aged <5 years of >50 deaths/1000 births or with more than 50,000 children’s deaths annually should make the introduction of PCV-7 a high priority for their immunization programs.

Rotavirus (RV) vaccines were successful in preventing severe rotavirus diarrhea and all-cause diarrhea (severe cases) (RR 0.59; 95% CI: 0.42, 0.83) [34]. Moreover,RV1 vaccine showed better results compared to RV5- (RR 0.20; 95% CI: 0.11, 0.35); [34].Efficacy and safety of three main types of rotavirus vaccines (bovine, human, and rhesus) demonstrated results varying from 22 to 89% to prevent one episode of rotavirus diarrhea, 11 to 44% to prevent one episode of all-cause diarrhea, and 43 to 90% to prevent one episode of severe rotavirus diarrhea [35]. Currently marketed rotavirus vaccines can prevent 74% (95% CI: 35, 90%) of rotavirus deaths and 47-57% of rotavirus hospitalizations [36].

A recent review on the effect of oral vaccines in terms of immunogenicity, indicated a greater than two folds rise in the vibriocidal antibody titres (RR 2.24, 95% CI: 1.32, 3.80); live oral vaccines showed a more significant rise as compared to killed oral vaccines [37]. A Review by Das et al. reported that cholera vaccinationwas associated with a 52% reduction in cholera incidence (RR 0.48, 95% CI: 0.35, 0.64) while, shigella vaccine had no impact on reducing the incidence of the disease (RR: 0.72, 95% CI: 0.37, 1.39) [37].

### Vitamin A supplementation from 6 months of age in Vitamin A deficient population

Vitamin A is a term used for a group of fat-soluble compounds required for the growth and differentiation of different body cells [38]. It also acts as an anti-oxidant agent and is especially involved in regeneration of epithelium of respiratory and gastrointestinal tracts [39]. Deficiency of vitamin A makes infant and children susceptible to infections [40]. Intervention with vitamin A in neonatal period and in children older than six months of age has shown reduction in all-cause mortality and cause specific mortality of diarrhea and measles [21,41].

Recent Cochrane and non-Cochrane reviews reported significant impact of vitamin A supplementation in reducing mortality due to any unspecified cause (RR 0.75; 95% CI: 0.65-0.87) [42]; (RR 0.76; 95% CI: 0.69-0.83) [43]; (RR 0.76; 95% CI: 0.69-0.83) [44] as well as mortality due to diarrhea: (RR 0.68; 95 % CI: 0.57-0.81) [42], (RR 0.72; 95% CI: 0.57-0.91) [43], (RR 0.72; 95% CI: 0.57-0.91) [44]. The reviews did not find any impact of vitamin A supplementation on mortality due to pneumonia, however mortality due to measles decreased (RR 0.50; 95% CI: 0.37-0.67) [43]. The review by Mayo-Welson et al. 2011 also reported reduced incidence of diarrhea: (RR 0.85; 95% CI: 0.82-0.87), vision problems (RR 0.32; 95% CI: 0.21-0.50), and xerophthalmia (RR 0.31; 95% CI: 0.22-0.45) [44] with supplementation.

### Management of severe acute malnutrition

Severe acute malnutrition (SAM) in children is defined as having a weight-for-height Z score of <3 according to WHO growth standards; visible wasting; or the presence of nutritional edema. It is estimated that malnutrition directly or indirectly is responsible for 35% of deaths occurring in children under five, so it is vital to correct malnutrition globally in order to decrease child mortality [45].

Implementation of the WHO guidelines (that includes early initiation of fluids, proper feeding, slow oral rehydration, broad-spectrum antimicrobial therapy and promptly managing all complications if present) showed reduction in mortality (RR 0.39; 95%CI: 0.28, 0.53) when compared with no guidelines. On the other hand, ready to use therapeutic food RUTF (a dry, solid diet without any addition of water so risk of bacterial contamination is eliminated) showed improvement in weight gain (g/kg/day) (Mean Difference (MD) 1.36; 95% CI: 0.33, 2.40) when compared with provision of corn flour in moderately malnourished children [46]. A recent review reported a reduction (ranged 3.4% to 35%) in case fatality rates among children who were treated in in-patient using the WHO protocol [47]. For community-based treatment of SAM, children given RUTF were 51% more likely to achieve nutritional recovery than the standard care group (RR 1.51; 95% CI: 1.04, 2.20) [47].

### Case management of childhood pneumonia

About 7.6 million children die every year before they reach their fifth birthday [48]. The main causes of these deaths are: acute respiratory infections, diarrhea, measles, malaria, malnutrition, or often a combination of these conditions. WHO and UNICEF launched a strategy, called Integrated Management of Childhood Illness (IMCI), to reduce deaths due to these manageable and preventable causes. IMCI is a strategy to reduce childhood mortality and morbidity from acute respiratory infections, diarrhea, measles, malaria, and malnutrition.

Systematic analysis by Theodoratou et al. 2010 found a significant impact of community case management with antibiotic on reducing ARI mortality (RR: 0.65; 95% CI: 0.52, 0.82) and all-cause mortality (RR: 0.79; 95% CI: 0.70, 0.88) among children 0-5 years [49]. A relatively older review also reported similar findings on reducing total mortality (RR 0.76; 95% CI: 0.67, 0.86) and pneumonia mortality (RR 0.64; 95% CI: 0.51, 0.80) among children 0-4 years of age [50]. However, a recent pooled analysis of community based interventions found a significant increase in care seeking (13%) and a signficant decrease in treatment failure rates (40%) for pneumonia [51]. The review also reported beneficial impact of community case management of pneumonia on reducing pneumonia specific mortality by 32% [51].

Among children with measles, vitamin A supplementation had a protective effect on reducing mortality (odds ratio (OR 0.39; 95% CI: 0.22, 0.66) [52]. Another review reported that a single dose of measles vaccine can reduce the incidence ofmeasles disease by 85% [95% CI: 83, 87) [53]. The stratified data from the same review showed that among children who were provided with vitamin A ( two doses of 200,000 IU for children >1 year and 100,000 IU for infants), measles mortality significantly reduced by 62% (RR 0.38; 95% CI: 0.18,0.81) [53].

Vitamin A as part of treatment for non-measles associated pneumonia for children above 6 months showed thatwhen a high-dose of vitamin A was given along with standard treatment of acute lower respiratory tract infection, no significant recovery was seen in children in developing countries aged from 1 month to 6 years [54]. Other reviews showed no significant reduction in mortality associated with pneumonia in children treated with vitamin A compared to those who were not (OR 1.29; 95% CI: 0.63, 2.66). Also, there was no significant difference in duration of hospital stay, and disease severity after supplementary high-dose vitamin A was significantly worse compared with placebo [55].

Grotto et al, on the other hand, indicated that vitamin A supplementation has no consistent overall protective effect on the incidence of diarrhea (RR 1.00; 95% CI: 0.94, 1.07) and that it slightly increases the incidence of respiratory tract infections (RR 1.08; 95% CI: 1.05, 1.11) [56]. Similarly, Chen et al. reported that most of the included studies found no significant effect of vitamin A on the incidence of acute LRTI, or prevalence of symptoms of acute LRTI [57]. Lamberti et al. indicated that pneumonia mortality was higher among infants who were not breastfed compared to exclusively breastfed infants of 0-5 months of age (RR 14.97; 95% CI: 0.67, 332.74) and among not breastfed compared to breastfed infants and young children 6-23 months of age (RR 1.92; 95% CI: 0.79, 4.68) [58].

### Case management of diarrhea

a. Preventive zinc supplementation in children

A number of intervention trials have been conducted in a variety of settings to assess the impact of preventive zinc supplementation on children’s health and development. The results of these studies are inconsistent, possibly because of differences in the underlying zinc status or other characteristics of the study populations or discrepancies in the research methods [59,60]. Previous reviews have shown that preventive zinc supplementation has a beneficial effect on reduction of incidence of diarrhea, pneumonia and other infectious diseases [61].

Zinc supplementation in children with diarrhea showed a decrease in diarrhea specific mortality (RR 0.81; 95% CI: 0.64,1.03), in pneumonia specific mortality (RR 0.86; 95% CI: 0.66, 1.12) and similar reductions in diarrhea and pneumonia specific morbidities [62].

b. Therapeutic zinc supplementation in children

Zinc deficiency is one of the leading risk factors for morbidity and mortality [63,64]. It is estimated that zinc deficiency is responsible for approximately 800,000 excess deaths annually among children under 5 years of age. The existing literature provides evidence of a beneficial effect of therapeutic zinc supplementation for the treatment of diarrhea shown to decrease the duration and severity of the diarrheal episode, diarrhea hospitalization rates and, in some studies, all-cause mortality [65].

Zinc supplementation may shorten the duration of diarrhea by around 10 hours (MD -10.44 hours; 95% CI: -21.13 to 0.25). It also reduces the number of children whose diarrhea persists until day seven (RR 0.73; 95% CI: 0.61 to 0.88), and the duration of persistent diarrhea shortens by around 16 hours (MD -15.84 hours; 95% CI: -25.43 to -6.24) [66].

c. ORS for diarrhea in children

Children who lose a large volume of liquid stool may develop moderate or severe dehydration; in the most severe cases this can lead to death. These children should be given rehydration therapy in order to restore the lost fluids and electrolytes. Oral rehydration solutions (ORS) have had a massive impact worldwide in reducing the number of deaths related to diarrhea. Most ORS is in the form of a sugar-salt solution, but over the years people have tried adding a variety of compounds (’glucose polymers’) such as whole rice, wheat, sorghum, and maize.

A review by Gregorio et al showed decreased unscheduled IV infusions (RR 0.75; 95% CI: 0.59-0.95) with polymer based ORS compared to glucose based ORS [67]. Hartling et al., on the other hand compared ORS therapy with intravenous therapy and reported significant risk difference intreatment failure (RD 4%; 95% CI: 1%, 7%); and non-significant impact on hospital stay (WMD -1.2; 95% CI: -2.38, 0.02). The same review reported 50 number needed to treat (NNT) forphelibitis: (95% CI: 25-100); and 33 for paralytic illeus(95% CI: 20, 100) [68]. When reduced osmolarity ORS was compared with WHO standard ORS, a reduction in unscheduled IV Infusion was observed (OR 0.59; 95% CI: 0.45, 0.79) [69].

Lenters et al. reported that the co-promotion of zinc and ORS indicated that mothers were 1.82 (95% CI 1.17, 2.85) times more likely to treat their child’s diarrhoea episode than mothers in the comparison group [70]. Mothers who were exposed to media and social marketing strategies through radio, television and cinema spots as well as community outreach activities and print materials were 2.05 (95% CI 0.78, 5.42) times more likely to use ORS to treat their child’s diarrhoea episode than mothers who were not exposed [70].

Vomiting due to acute gastroenteritis limits the success of oral rehydration leading to an increased use of intravenous rehydration, need for prolonged emergency department stays and hospitalizations. Das et al. reported that use of antiemetic in diarrhoea was associated with a significant 54% reduction in the incidence of vomiting (RR 0.46; 95% CI: 0.35, 0.61) and 54% reduction in the incidence of hospitalization (RR: 0.46, 95% CI: 0.29, 0.74) [71].

d. Treatment of dysentery in children

Shigellosis occurs predominantly in LMICs and is most common where overcrowding and poor sanitation exist. Shigellosis is a bacterial infection of the colon that causes diarrhea and can lead to death. Dysentery is frequent mucoid or bloody stools. When dysentery is caused by Shigella it is called Shigella dysentery. The intention of giving antibiotics in shigellosis is to speed recovery, reduce the seriousness of the disease, and reduce the length of time patients are infective. However, some antibiotics can have serious side effects while others may not be effective against the Shigella bacteria.

Reduced episodes of diarrhoea at follow-up were seen with Furazolidone versus no drug (RR 0.21; 95% CI: 0.09, 0.48) as well as with cotrimoxazole versus no drug (RR 0.30; 95% CI: 0.15, 0.59) [72]. However, treatment with one of the three WHO-recommended antibiotics (ciprofloxacin, ceftriaxone and pivmecillinam) resulted in a clinical failure rate of 0.1% (95% CI: −0.2, 0.5%) [73]. On the other hand, when two different antibiotics (Pivmecillinam and Ciprofloxacin) were used it resulted in 82% reduction in clinical failure (RR 0.18; 95% CI: 0.10, 0.33). While Pivmecillinam, Ciprofloxacin and Ceftriaxone reported 96% reduction in bacteriological failure of (RR 0.04; 95% CI: 0.01, 0.12) [74].

f. Treatment of cholera in children

Cholera is caused by ingesting food or water containing the bacterium Vibrio cholera. The disease can spread rapidly among populations lacking access to safe water and adequate sanitation facilities. Symptoms of cholera include acute watery diarrhea, vomiting, and severe dehydration, which can lead to death within 24 hours if left untreated [75]. Because cholera is associated with significant electrolyte loss especially among children, the use of ORS with reduced sodium levels may place patients at a greater risk for developing biochemical hyponatremia (low blood sodium levels <130mmol/L) [76], which can result in severe illness including seizures, respiratory arrest, coma (symptomatic hyponatremia), and even death.

For glucose-based ORS, biochemical hyponatraemia (blood sodium levels <130mmol/L) was more common with ORS ≤270 (RR 1.67, 95% CI 1.09, 2.57), while a higher level of severe biochemical hyponatraemia (blood sodium levels <125mmol/L) in the same group was not significant (RR 1.58, 95% CI 0.62, 0.04). In the ORS ≤270 group (rice base ORS) , duration of diarrhoea was shorter (MD -11.42 hours, 95% CI -13.80, -9.04) [77]. The antibiotic treatment of cholera showed 63% reduction in clinical failure (RR 0.37; 95% CI: 0.19, 0.71) [74]**.**

## Discussion

The threat to child survival continues to be of grave concern. Therefore, this paper summarizes all the essential preventive and therapeutic child health interventions for preventing illnesses and improving child survival. The importance of these interventions have been addressed in previous publications [78-82] and more recently in the Lancet Neonatal Series [83]. However this review progresses further by collating and analyzing existing evidence to assist health professionals and policy makers reduce the current burden of child morbidity and mortality.

What cannot be stressed enough is the importance of exclusive breastfeeding of children till at least the age of 6 months; this ensures proper growth and development of the child. To prevent growth faltering or a decline in the child’s motor and physical development, continued breastfeeding is an intervention that is being implemented in many regions where mothers may be substituting poor quality weaning foods in place of breastfeeding. It should also be noted that poverty and poor water and sanitary conditions are some of the underlying causes of poor child growth and development and infection- specific mortality; and that, breastfeeding, alone, cannot contribute in improving health and survival. Therefore, promotion of breastfeeding should be implemented in conjunction with a larger strategy that includes promotion of complementary feeding, improved water and sanitation, and better health care services.

Interventions to target the main causes of under-five mortality including pneumonia, diarrhea, malaria and under nutrition is the key to further success in bringing down child mortality. Interventions including antibiotics, ITNs, and improved family care need to be implemented to further reduce child morbidity and mortality rates. In endemic regions or tropical areas, malarial prophylaxis is vital; the expanded program on immunization (EPI) has been and will continue to be one of the most cost effective methods of improving mortality and morbidity rates. Evidence suggested that ITN use can reduce child mortality by 18% and 5.5 lives can be saved annually for every 1000 children between 1-59 months protected. In areas with seasonal transmission, IPT showed a dramatic decrease in clinical malaria during intervention, as well as rates of parasitemia post intervention. However, long-term follow-up studies are needed for efficacy of IPT use in children, including its safety.

Preventive zinc supplementation shows promising benefits in reducing diarrhea specific mortality but further supporting data is required. On the other hand, therapeutic zinc supplementation has a beneficial effect on reducing duration and severity of acute and persistent diarrhea. The results indicate that scaling up the use of zinc in health systems is feasible particularly in LMICs. However, the evidence for beneficial effect of zinc in the treatment of pneumonia, malaria and tuberculosis is limited and needs further evaluation. There are a number of available opportunities to deliver zinc supplementation as one component of programs to prevent micronutrient deficiencies and to address other nutrition and health needs of infants and children. Efforts are needed to test these delivery mechanisms and evaluate their potential for providing cost-effective preventive zinc supplementation to high-risk target groups on a large scale.

Multi-country evaluation of implication of IMCI has shown that it has been very effective in reducing the mortality and morbidity from ARI, diarrhea, measles and malnutrition. It is cost effective and applicable on larger scale. Considering the strengths of the IMCI strategy and the existing commitments and investments by countries, the IMCI strategy, should be continued and expanded, as part of a broader investment approach to improve child health outcomes. Vitamin A supplementation in children after six months of age reduces all-cause mortality and diarrhea specific and measles specific mortality. There is a very limited evidence for protective effect of vitamin A for prevention of pneumonia specific mortality and morbidity. Due to its beneficial effect on all-cause and diarrhea-specific mortality, routine vitamin A supplementation in this age group is recommended.

As we move closer to the deadline for the target of the MDGs, a closer look at the existing programs and interventions was necessary to evaluate where opportunities for improvement lie. This paper is a comprehensive and thorough summary of essential child health interventions and can be used by health professionals and decision makers from the field. The strength of this paper lies in the fact that it includes and summarizes evidence from all recent Cochrane and non-Cochrane reviews on child health interventions. However, at times the quality of all included studies within the review could not be ensured which limits the quality of the data obtained.

## Conclusion

High rates of child mortality are unquestionably linked to inadequate and poor quality health services available in the early years of life both to mother and child. In a majority of countries, especially low-income countries, the availability of health care services, vaccination programs and other vital amenities are inadequate, of poor quality, and require positive transformation. Because a major proportion of child deaths occur in LMICs, the need for appropriate education and awareness is vital prior to establishing interventions. Empowerment of women, removing barriers to accessibility to health care services, increased education and awareness in communities, and shifting the focus to evidence based interventions may help inculcate healthy practices among mothers and improve child survival rates. Appropriate culturally sensitive education and awareness provided to the communities, followed by timely implementation of discussed interventions which can be infused with existing healthcare practices, will definitely bring the required improvement in child health and survival.

## Competing interests

We do not have any financial or non-financial competing interests for this review.

## Peer review

The reviewer reports for this article can be found in Additional File 1.

## Supplementary Material

Additional file 1 **Peer review reports are included in additional file**1. Click here for file

## Declarations

This article has been published as part of *Reproductive Health* Volume 11 Supplement 1, 2014: Essential intervention for maternal, newborn and child health. The full contents of the supplement are available online at http://www.reproductive-health-journal.com/supplements/11/S1. Publication charges for this collection were funded by the Partnership for Maternal, Newborn & Child Health (PMNCH).
