# Supplementary material for: Essential interventions for child health
Source: Reprod Health. 2014 Aug 21;11(Suppl 1):S4. doi: 10.1186/1742-4755-11-S1-S4 (PMC4145856; doi:10.1186/1742-4755-11-S1-S4)
Supplement: Additional file 1 — Peer review reports are included in additional file1. [file 1742-4755-11-S1-S4-S1.pdf]

Referee's comments to the authors– this sheet WILL be seen by the author(s) and published with the article

Title	**Essential Interventions for child health**
Author(s)	Zohra S Lassi, Dania Mallick, Jai K Das, Lekho Mal, Rehana A Salam, Zulfiqar A Bhutta
Referee's name	Clara Menéndez

When assessing the work, please consider the following points, where applicable:

1. Is the question posed by the authors new and well defined?
2. Are the methods appropriate and well described, and are sufficient details provided to replicate the work?
3. Are the data sound and well controlled?
4. Does the manuscript adhere to the relevant standards for reporting and data deposition?
5. Are the discussion and conclusions well balanced and adequately supported by the data?
6. Do the title and abstract accurately convey what has been found?
7. Is the writing acceptable?

Please make your report as constructive and detailed as possible in your comments so that authors have the opportunity to overcome any serious deficiencies that you find and please also divide your comments into the following categories:

- Major Compulsory Revisions (which the author must respond to before a decision on publication can be reached)
- Minor Essential Revisions (such as missing labels on figures, or the wrong use of a term, which the author can be trusted to correct)
- Discretionary Revisions (which are recommendations for improvement but which the author can choose to ignore)

Where possible please supply references to substantiate your comments.

When referring to the manuscript please provide specific page and paragraph citations where appropriate.

General comments:
This manuscript answers a very relevant and critical global health issue using interesting published data. However, in my opinion it is too long and poorly constructed. It could be better summarized and organized. It is difficult for the reader to keep the track of the findings. There is repetitive information throughout the text, and key information on methods of the literature review has been omitted.
Author should indicate the most important gaps, inconsistencies and/or controversies in the literature that the current study will address. The author should also explain the study's ultimate goal and main contribution.
Discussion and conclusions are often mixed up with the results of the different interventions, whereas overall conclusion section is very vague and disproportionately short with the rest of the text. It is strongly advised to structure the discussion by the key findings and again, to review the English writing style.
Discretionary revisions

Major compulsory revisions:
The title does is not representative of what the manuscript is about. Does not indicate what was done, I would suggest to modify it. (Ex; "Adressing/reviewing/comparing essential interventions for child health")

The question addressed is not clearly defined, nor in the abstract or the introduction. We assume it is to identify the most effective interventions improving child's health and survival, but it is not stated anywhere in the text. I think the motivations for this study need to be made clearer

The main objective of the revision is not mentioned in the manuscript. I suggest authors' to briefly describe what is innovative about this review why it is important and its usefulness (not focusing on reducing child mortality, which is obvious, but on purpose of reviewing and comparing interventions).

Some discussion on interventions is presented in the introduction, while it should be left for the conclusion/discussion section

The manuscript lacks a consistent formal structure. In the results the author introduces the rationale of each intervention and then presents the results and its impact on child outcomes. I suggest not including background information on the disease on the results section, neither conclusions nor personal comments. Results are not clearly presented. The author should expose concisely the studies or trials reviewed and main findings. There is lack of consistency in the exposure of results found in the different interventions.

The most relevant studies mentioned should be very briefly explained when presenting the results. There are no comments on limitations neither of the manuscript nor of each of the studies evaluated.

Conclusion section should be a more intervention-specific and in-depth discussion.

Minor essential revisions:
Please find minor revisions to following sections;
<u>Abstract</u>
Avoid starting phrases with numbers (see second phrase of the abstract, or second phrase of second pharagraph in page 3)
<u>Introduction</u>
Revise reference number 1, as the paper cited is on causes of maternal mortality.
Avoid personal opinions, not supported by referenced evidence, such as “empowerment of women, removing barriers, increased education….will help improve mortality rates”.
In page 2 rephrase last phrase of paragraph 3.
In third phrase of fourth paragraph when mentioning cost-effective interventions for malaria, antibiotics should be substituted by antimalarials.
<u>Results</u>

- Exclusive breastfeeding. Evaluate changing this subtitle to “Promotion of breastfeeding”.
  Second phrase of first paragraph should be referenced.
  Last paragraph should be in discussion/conclusions section
- Complementary Feeding support and Education strategies. Consider change in subtitle, ex; Education strategies on complementary feeding support.
  In second paragraph; error in Hb value, out of range.
  Last paragraph should be in discussion/conclusions section.
  Define acronyms HAZ, SM, WAZ.
  Second set of results from “However, based on sub group analysis..” needs to be referenced.
  Last paragraph should be included in discussion/conclusions section.
- Prevention and management of childhood malaria
  Phrases like “Malaria is a major killer of children. In Africa , around 66, 000 children die…” should not be included in the results, and “There is need for large-scale implementation of this intervention in malaria endemic areas” should be in the discussion/conclusions section.
- Malaria prophylaxis in children:
  Further on in you address malaria IPT in HIV pregnant women (not consistent with subtitle). The inclusion of this intervention is not fully explained. Consider that SP is contraindicated in HIV infected pregnant women in antiretrovirals.
- Comprehensive care of children infected or exposed to HIV infection
  First phrase should be included in the introduction rather than in the results section.
  Early diagnosis and antiretroviral treatment on HIV infected children reduces child mortality, Evaluate if it should be commented.
  Last paragraph on HIV guideline are not appropriate in the results section
- Promote and provide routine immunization and vaccination in infants/children.
  First paragraph would suit better in the introduction rather than in results section.
  Define acronym WHOEPI.
  Last paragraph is not appropriate in results section. Evaluate including it in discussin/conclusion section.
- Vitamin A supplementation from 6 months of age in vitamin A deficient population
  First paragraph would suit better in the introduction rather than in results section.
  Last paragraph is not appropriate in results section. Evaluate including it in discussin/conclusion section
- Case management of childhood pneumonia
  First paragraph is more appropriate for introduction. It is general for all diseases not just pneumonia. **le**
  Four last paragraphs should be in previous sub-section on vit A.
  Define acronym LRTI.
- Case management of diarrhea
  Too long and inappropriate introduction of each intervention for a results section, together with conclusive comments which will be better included in discussion section.

Title	**Essential Interventions for child health**
Author(s)	Zohra S Lassi, Dania Mallick, Jai K Das, Lekho Mal, Rehana A Salam, Zulfiqar A Bhutta
Referee’s name	Tom Lissauer

When assessing the work, please consider the following points, where applicable:

1. Is the question posed by the authors new and well defined?
2. Are the methods appropriate and well described, and are sufficient details provided to replicate the work?
3. Are the data sound and well controlled?
4. Does the manuscript adhere to the relevant standards for reporting and data deposition?
5. Are the discussion and conclusions well balanced and adequately supported by the data?
6. Do the title and abstract accurately convey what has been found?
7. Is the writing acceptable?

Please make your report as constructive and detailed as possible in your comments so that authors have the opportunity to overcome any serious deficiencies that you find and please also divide your comments into the following categories:

- Major Compulsory Revisions (which the author must respond to before a decision on publication can be reached)
- Minor Essential Revisions (such as missing labels on figures, or the wrong use of a term, which the author can be trusted to correct)
- Discretionary Revisions (which are recommendations for improvement but which the author can choose to ignore)

Where possible please supply references to substantiate your comments.

When referring to the manuscript please provide specific page and paragraph citations where appropriate.

General comments: This is a detailed review of the child health interventions which have been shown to globally improve outcomes. It provides a succinct summary of the current evidence to support strategies, backed up by a table listing the reviews of the interventions considered. It will be a helpful resource for anyone wanting a distillation of the interventions shown to improve health outcomes globally.

Major compulsory revisions:

Minor essential revisions: Something seems to have gone wrong with the introductory section. The introduction should contain background information, setting the scene, but it also includes recommendations based on the results of this paper. Although the abstract explains the purpose of the paper, ie it “reviews the interventions from the child health which if implemented properly and according to guidelines has found to improve child health outcomes, and reduce morbidity and mortality

(continue on the next sheet)

Continued:
this has been omitted from the paper. There clearly needs to be a section on the purpose of the paper and something on the methodology including reference to Table 1 in the text.
There are also typos such as “phelibiltis” (page 10) and editing of some sentences is required eg the one quoted above is not grammatically correct and there are others eg “While Pivmecillinam, Ciprofloxacin and Ceftriaxone reported on bacteriological failure showing a reduction of 96%” on page 11.

Referee's comments to the authors– this sheet WILL be seen by the author(s) and published with the article

Title	**Essential Interventions for child health**
Author(s)	Zohra S Lassi, Dania Mallick, Jai K Das, Lekho Mal, Rehana A Salam, Zulfiqar A Bhutta
Referee's name	Clara Menéndez

When assessing the work, please consider the following points, where applicable:

1. Is the question posed by the authors new and well defined?
2. Are the methods appropriate and well described, and are sufficient details provided to replicate the work?
3. Are the data sound and well controlled?
4. Does the manuscript adhere to the relevant standards for reporting and data deposition?
5. Are the discussion and conclusions well balanced and adequately supported by the data?
6. Do the title and abstract accurately convey what has been found?
7. Is the writing acceptable?

Please make your report as constructive and detailed as possible in your comments so that authors have the opportunity to overcome any serious deficiencies that you find and please also divide your comments into the following categories:

- Major Compulsory Revisions (which the author must respond to before a decision on publication can be reached)
- Minor Essential Revisions (such as missing labels on figures, or the wrong use of a term, which the author can be trusted to correct)
- Discretionary Revisions (which are recommendations for improvement but which the author can choose to ignore)

Where possible please supply references to substantiate your comments.

When referring to the manuscript please provide specific page and paragraph citations where appropriate.

General comments:
This manuscript answers a very relevant and critical global health issue using interesting published data. However, in my opinion it is too long and poorly constructed. It could be better summarized and organized. It is difficult for the reader to keep the track of the findings. There is repetitive information throughout the text, and key information on methods of the literature review has been omitted.
Revisited the text and removed redundant text. Methods section added.

Author should indicate the most important gaps, inconsistencies and/or controversies in the literature that the current study will address. The author should also explain the study's ultimate goal and main contribution.
Added
Discussion and conclusions are often mixed up with the results of the different interventions, whereas overall conclusion section is very vague and disproportionately short with the rest of the text. It is strongly advised to structure the discussion by the key findings and again, to review the English writing style.
Discussion added and whole paper is now checked for English editing.
Discretionary revisions

Major compulsory revisions:
The title does is not representative of what the manuscript is about. Does not indicate what was done, I would suggest to modify it. (Ex; "Adressing/reviewing/comparing essential interventions for child health")
We see no harm in adding the word "Reviewing". However, this title is in line with other titles in the supplement.

The question addressed is not clearly defined, nor in the abstract or the introduction. We assume it is to identify the most effective interventions improving child's health and survival, but it is not stated anywhere in the text. I think the motivations for this study need to be made clearer
Added the word essential in abstract. However in intro added a paragraph (last pra on introduction) on describing the aim of the paper

The main objective of the revision is not mentioned in the manuscript. I suggest authors' to briefly describe what is innovative about this review why it is important and its usefulness (not focusing on reducing child mortality, which is obvious, but on purpose of reviewing and comparing interventions).
Added in the last paragraph of introduction

Some discussion on interventions is presented in the introduction, while it should be left for the conclusion/discussion section
We have now moved some section from Intro to the discussion section.

Study design and methods are not described. We do not know the procedures of the literature review or the sources of information, neither the inclusion criteria for the reviewed articles. There are no sufficient details to replicate this work.
Added the methods section.

The manuscript lacks a consistent formal structure. In the results the author introduces the rationale of each intervention and then presents the results and its impact on child outcomes. I suggest not including background information on the disease on the results section, neither conclusions nor personal comments. Results are not clearly presented. The author should expose concisely the studies or trials reviewed and main findings. There is lack of consistency in the exposure of results found in the different interventions.
Revisited. Removed the repetitive sentences particularly the summary paragraphs.

The most relevant studies mentioned should be very briefly explained when presenting the results. There are no comments on limitations neither of the manuscript nor of each of the studies evaluated.
We have now added them in the discussion section

Conclusion section should be a more intervention-specific and in-depth discussion.
We have now added a detailed discussion section. And revisited conclusion.

Minor essential revisions:
Please find minor revisions to following sections;
Abstract
Avoid starting phrases with numbers (see second phrase of the abstract, or second phrase of second pharagraph in page 3)
Corrected

Introduction
Revise reference number 1, as the paper cited is on causes of maternal mortality. Corrected
Avoid personal opinions, not supported by referenced evidence, such as "empowerment of women, removing barriers, increased education….will help improve mortality rates". Removed.
In page 2 rephrase last phrase of paragraph 3. Rephrased.
In third phrase of fourth paragraph when mentioning cost-effective interventions for malaria, antibiotics should be substituted by antimalarials. Removed.
Results

- Exclusive breastfeeding. Evaluate changing this subtitle to "Promotion of breastfeeding". Changed
  Second phrase of first paragraph should be referenced. Removed.
  Last paragraph should be in discussion/conclusions section moved
- Complementary Feeding support and Education strategies. Consider change in subtitle, ex; Education strategies on complementary feeding support. Changed
  In second paragraph; error in Hb value, out of range.
  Last paragraph should be in discussion/conclusions section. moved
  Define acronyms HAZ, SM, WAZ. Defined.
  Second set of results from "However, based on sub group analysis.." needs to be referenced.
  Last paragraph should be included in discussion/conclusions section. moved

Prevention and management of childhood malaria

- Phrases like “Malaria is a major killer of children. In Africa , around 66, 000 children die…” should not be included in the results, and “There is need for large-scale implementation of this intervention in Malaria prophylaxis in children:malaria endemic areas” should be in the discussion/conclusions section. Removed and for the second suggestion we moved the sentence to discussion.

Comprehensive care of children infected or exposed to HIV infection

First phrase should be included in the introduction rather than in the results section. Removed
Early diagnosis and antiretroviral treatment on HIV infected children reduces child mortality, Evaluate if it should be commented. Couldn’t find this.
Last paragraph on HIV guideline are not appropriate in the results section moved

Promote and provide routine immunization and vaccination in infants/children.

First paragraph would suit better in the introduction rather than in results section. Well, we were told to keep intro brief and this format is in line with other papers from the same series.
Define acronym WHOEPI. Removed
Last paragraph is not appropriate in results section. Evaluate including it in discussin/conclusion section. moved

Vitamin A supplementation from 6 months of age in vitamin A deficient population

First paragraph would suit better in the introduction rather than in results section. Well, we were told to keep intro brief and this format is in line with other papers from the same series.

Last paragraph is not appropriate in results section. Evaluate including it in discussin/conclusion section moved

Case management of childhood pneumonia

First paragraph is more appropriate for introduction. It is general for all diseases not just pneumonia. Well, we were told to keep intro brief and this format is in line with other papers from the same series.

Four last paragraphs should be in previous sub-section on vit A. This is how these interventions were treated in the Main PMNCH Essential intervention document.
Define acronym LRTI.

Case management of diarrhea

Too long and inappropriate introduction of each intervention for a results section, together with conclusive comments which will be better included in discussion section. Revisited and moved some section to discussion and others in line with the format in other papers.

Referee’s comments to the authors– this sheet WILL be seen by the author(s) and published with the article

Title	**Essential Interventions for child health**
Author(s)	Zohra S Lassi, Dania Mallick, Jai K Das, Lekho Mal, Rehana A Salam, Zulfiqar A Bhutta
Referee’s name	Tom Lissauer

When assessing the work, please consider the following points, where applicable:

1. Is the question posed by the authors new and well defined?
2. Are the methods appropriate and well described, and are sufficient details provided to replicate the work?
3. Are the data sound and well controlled?
4. Does the manuscript adhere to the relevant standards for reporting and data deposition?
5. Are the discussion and conclusions well balanced and adequately supported by the data?
6. Do the title and abstract accurately convey what has been found?
7. Is the writing acceptable?

Please make your report as constructive and detailed as possible in your comments so that authors have the opportunity to overcome any serious deficiencies that you find and please also divide your comments into the following categories:

- Major Compulsory Revisions (which the author must respond to before a decision on publication can be reached)
- Minor Essential Revisions (such as missing labels on figures, or the wrong use of a term, which the author can be trusted to correct)
- Discretionary Revisions (which are recommendations for improvement but which the author can choose to ignore)

Where possible please supply references to substantiate your comments.

When referring to the manuscript please provide specific page and paragraph citations where appropriate.

General comments: This is a detailed review of the child health interventions which have been shown to globally improve outcomes. It provides a succinct summary of the current evidence to support strategies, backed up by a table listing the reviews of the interventions considered. It will be a helpful resource for anyone wanting a distillation of the interventions shown to improve health outcomes globally.

Major compulsory revisions:

Minor essential revisions: Something seems to have gone wrong with the introductory section. The introduction should contain background information, setting the scene, but it also includes recommendations based on the results of this paper. Although the abstract explains the purpose of the paper, ie it "reviews the interventions from the child health which if implemented properly and according to guidelines has found to improve child health outcomes, and reduce morbidity and mortality "rates. **this does**

Revisited and moved and shuffled text in Intro. Added Discussion section.

Continued:
this has been omitted from the paper. There clearly needs to be a section on the purpose of the paper and something on the methodology including reference to Table 1 in the text.
There are also typos such as "phelibiltis" (page 10) and editing of some sentences is required eg the one quoted above is not grammatically correct and there are others eg "While Pivmecillinam, Ciprofloxacin and Ceftriaxone reported on bacteriological failure showing a reduction of 96%" on page 11.
Corrected those and many others.
